# Editorial: Role of gamma delta T cells in cancer immunotherapy

**DOI:** 10.3389/fimmu.2026.1870909

**Published:** 2026-05-15

**Authors:** Kawaljit Kaur

**Affiliations:** ImmuneLink, LLC, San Diego, CA, United States

**Keywords:** cancer, CAR, gamma delta (γδ) T cells, immunotherapy, therapeutics

Gamma delta (γδ) T cells are a unique type of T lymphocyte with specialized γ and δ T-cell receptor (TCR) chains, bridging innate and adaptive immunity (1). First discovered in the 1980s, by 1987, they were identified as CD3-associated molecules forming a γ and δ chain heterodimer (2–4). They develop in the thymus from the same progenitors as αβ T cells, emerging early from double-negative thymocytes under the influence of Notch signaling and interleukin-7 (IL-7) (5). γδ T cells account for about 1–10% of T cells in human peripheral blood but are more often found in epithelial and mucosal tissues like the skin, gut, lungs, and reproductive tract (6, 7). The main subsets—Vδ1, Vδ2 (mostly Vγ9Vδ2), and Vδ3—differ in location, recognition targets, and function. Vδ1 T cells live in tissues like the gut, liver, skin, and spleen, recognizing stress ligands like MICA/B and glycolipids via CD1d (5, 8). Vδ1 T cells have strong cytotoxic abilities, express NK receptors such as NKG2D, and resist activation-induced cell death for long-lasting anti-tumor effects (9). Vγ9Vδ2 T cells, found mainly in the blood, respond to phosphoantigens like IPP from disrupted tumor mevalonate pathways and are activated by butyrophilin complexes BTN3A1 and BTN2A1 (10, 11). These cells kill target cells, release cytokines like IFN-γ and TNF-α, and can mediate ADCC through CD16 (12, 13). Vδ3 T cells, less understood, are present in the liver and gut, respond to glycolipids and oxidative stress presented by CD1d, and may also appear in the blood during leukemia or viral infections (14, 15).

γδ T cells can directly kill tumor cells, enhance immune activity through cytokines, and support adaptive responses (16, 17). Unlike αβ T cells, they detect antigens without depending on MHC, letting them broadly target tumors, respond quickly, and take on diverse roles in cancer defense (5, 18). Therefore, γδ T cells show great potential in cancer immunotherapy, particularly for tumors with low MHC expression or high heterogeneity that pose challenges to traditional treatments (19, 20). Tumor destruction occurs via granule exocytosis, releasing perforin and granzyme B, triggering death receptor pathways like TRAIL and FasL, and carrying out ADCC by binding CD16 to antibody-coated tumor cells (16, 21–23). They also release pro-inflammatory cytokines such as IFN-γ and TNF-α, shaping the tumor microenvironment and stimulating other immune cells (22, 24).

Advances in genetic engineering, cell expansion, and clinical trials are driving the development of next-generation treatments, especially for solid tumors and cancers that resist standard T cell therapies (25). Both non-engineered and engineered γδ T cell treatments are under clinical investigation, highlighting their versatility in cell-based and engager approaches (26). Non-engineered methods use ex vivo expanded autologous or allogeneic γδ T cells (mainly Vγ9Vδ2), stimulated by aminobisphosphonates (like zoledronate or pamidronate) and cytokines (like IL-2) to boost their expansion and activation before infusion (26–28). Clinical trials for both hematologic and solid tumors have shown that γδ T cell therapies are safe, can be produced at scale, and lead to some measurable responses, though complete remissions remain uncommon (26, 29). Phase 1 trials using ex vivo expanded donor-derived allogeneic γδ T cells after hematopoietic cell transplantation in high-risk acute myeloid leukemia patients reported encouraging safety results, with no cases of cytokine release syndrome (CRS), immune neurotoxicity (ICANS), or graft-versus-host disease (GVHD) (30, 31). Early signs of effectiveness include sustained complete remission (CR) and minimal residual disease (MRD) negativity (32). *In vivo* activation of γδ T cells can be achieved with aminobisphosphonates, synthetic phosphoantigens, and cytokines like IL-2, to stimulate endogenous Vγ9Vδ2 T cells (33, 34). Although this method faces challenges like cell exhaustion and only moderate efficacy, it’s still a useful complement. Bispecific γδ T cell engagers—synthetic molecules that link γδ TCRs to tumor antigens—can redirect and activate γδ T cells at tumor sites to boost their tumor-killing ability, with examples targeting Her2, CD40, or CD123 (35–37). The development of banked γδ T cell products for universal off-shelf use offers a promising way to cut costs and improve access (38). Genetic modifications address challenges like limited expansion, persistence, and tumor targeting (25). CAR-γδT cells, designed with chimeric antigen receptors to recognize tumor antigens, add greater precision and potency (39, 40). Preclinical and early clinical studies show promising safety and tumor-killing potential, including engineered Vδ1 CAR-T cells and γδ T cells derived from induced pluripotent stem cells (iPSCs) (41, 42). TCR gene transfer introduces high-affinity γδ TCRs into T cells to enhance tumor detection and anti-tumor effects (43). CRISPR/Cas9 edits remove inhibitory receptors to prevent exhaustion or add cytokine genes like IL-15 to boost cell expansion and persistence (44, 45).

Although notable progress has been made, developing γδ T cell therapies still faces hurdles like limited *in vivo* expansion, post-transfer persistence, and the immunosuppressive tumor microenvironment (TME) that hinders effectiveness (19, 20, 46, 47). The diversity and adaptability of γδ T cell subsets make consistent results challenging, and knowledge of γδ TCR ligands and activation hierarchies—particularly for subsets such as Vδ1 and Vδ3—remains incomplete (5). Current strategies focus on improving γδ T cell persistence and functional activation within the TME (46). Early clinical data indicate good tolerance, manageable side effects, and sustained immune recovery, supporting further dose-escalation and efficacy trials (30, 31). Engineering approaches can enhance therapeutic precision and resilience, while combining these treatments with checkpoint inhibitors, monoclonal antibodies (to leverage ADCC), chemotherapy, radiotherapy, or other immunomodulators could boost outcomes (48, 49). Innovative delivery systems, like nanoparticle-encapsulated agonists, show promise for optimizing pharmacodynamics and minimizing systemic toxicity (49–51). Clinical trials in both hematologic cancers and solid tumors are moving rapidly to refine dosing, persistence, and overall effectiveness (30, 31).

This Research Topic highlighted recent advancements in γδ T cell-based immunotherapies, approaches to optimize culture conditions for γδ T cell expansion, methods to rejuvenate exhausted γδ T cells, and strategies to boost the development and anti-cancer capabilities of CAR-γδ T cells. Oberg et al. have introduced “Evobodies,” a new type of biologic with a wide therapeutic range and strong activity against FOLR1-positive cells, while sparing healthy, FOLR1-negative tissue. The study points out that Evobodies are the first of their kind for treating solid tumors and show great promise for future clinical development. Chauvet et al. reviewed the current knowledge on Vγ9Vδ2 T cell exhaustion and looked into various ways to revive these cells for research, ranging from *ex vivo* collection and *in vivo* mouse models to advanced *in vitro* experiments. Morandi et al. investigated the potential of cytotoxic γδ T and NK cells (GADEKILL) as a new immunotherapy approach for high-risk neuroblastoma (HR-NB). Pinot et al. explored how adding human serum to cultures affects γδ T cells and looked at what this means for adoptive cell therapy. They compared the growth, characteristics, and functions of γδ T cells in serum-free versus serum-containing media. Their findings suggest that fine-tuning culture conditions for γδ T cell expansion could boost the clinical use of this approach and lead to better treatment outcomes for cancer patients. Kelm et al. explored how Blinatumomab (BLN) affects the effector functions of ex vivo-expanded healthy donor-derived γδ T cells compared to αβ T cells. Their *in vitro* results show subset-specific BLN responses and support the idea that ex vivo-expanded Vγ9Vδ2 γδ T cells may enhance BLN-mediated cytotoxicity, especially when CD19 density is high and target burden is low. These findings offer a mechanistic basis for further testing of γδ T-cell/BLN combination strategies in patient-derived models and clinical trials.

Wang et al. provided an overview and shared insights into the progress and future potential of γδ CAR-T cells. Pinot et al. presented a strategy to enhance CAR transgene expression in γδ T cells using a Baboon-pseudotyped lentiviral vector (BaEV-LV), in comparison to the conventional vesicular stomatitis virus G protein (VSV-G) LVs. BaEV-LV significantly increased the transduction efficiency of CAR-modified γδ T cells while preserving the composition and phenotype characteristic of unmodified γδ T cells. These BaEV-LV CAR γδ T cells exhibited markedly stronger cytotoxicity against B7H3-expressing tumor cells in both 2D and 3D *in vitro* models. This advancement represents a substantial step forward in CAR-γδ T cell engineering, offering a promising avenue for cancer immunotherapy by integrating the distinctive properties of Vγ9Vδ2 T cells with the precision of CAR technology. The method is compatible with automated closed systems such as the CliniMACS Prodigy^®^, supporting GMP-compliant production for clinical trials and enhancing the translational potential of these engineered cells for next-generation γδ T cell-based therapies.

## Summary

γδ T cells bridge innate and adaptive immunity, reacting quickly to stressed, infected, or abnormal cells through unique antigen recognition that bypasses traditional MHC pathways. Advances in molecular markers and single-cell technologies have deepened understanding of their development, diversity, and functions, opening doors to targeted cancer therapies for specific γδ T cell subsets or stages. Current clinical trials tap into their MHC-independent detection of tumor-associated and stress ligands to promote tumor killing, cytokine release, and immune modulation. Non-engineered approaches aim to expand these cells *in vivo* or ex vivo with defined activation cues, while engineered methods use CARs, TCR gene transfer, bispecific engagers, and genetic editing to tackle issues like persistence and immunosuppression. The ultimate goal is to deliver effective, ready-to-use immunotherapies for both blood and solid cancers. While early signs are encouraging, challenges such as tumor microenvironment resistance, expansion efficiency, and cell plasticity remain key research targets. Recent advancements are enabling sustained activation and growth, making large-scale γδ T cell expansion achievable and enhancing their potential as a promising therapeutic platform for cancer and other diseases.
